# Nanoporous Polymer Films of Cyanate Ester Resins Designed by Using Ionic Liquids as Porogens

**DOI:** 10.1186/s11671-017-1900-8

**Published:** 2017-02-17

**Authors:** Alexander Fainleib, Alina Vashchuk, Olga Starostenko, Olga Grigoryeva, Sergiy Rogalsky, Thi-Thanh-Tam Nguyen, Daniel Grande

**Affiliations:** 1 Institute of Macromolecular Chemistry, National Academy of Sciences of Ukraine, 48, Kharkivske shose, 02160 Kyiv, Ukraine; 2 Institute of Bioorganic Chemistry and Petrochemistry, National Academy of Sciences of Ukraine, 50, Kharkivske shose, 02160 Kyiv, Ukraine; 3 Institut de Chimie et des Matériaux Paris-Est, UMR 7182 CNRS – Université Paris-Est Créteil Val-de-Marne, 2, rue Henri Dunant, 94320 Thiais, France

**Keywords:** Cyanate ester resins, Nanoporous thermosetting films, Ionic liquids

## Abstract

Novel nanoporous film materials of thermostable cyanate ester resins (CERs) were generated by polycyclotrimerization of dicyanate ester of bisphenol E in the presence of varying amounts (from 20 to 40 wt%) of an ionic liquid (IL), i.e., 1-heptylpyridinium tetrafluoroborate, followed by its quantitative extraction after complete CER network formation. The completion of CER formation and IL extraction was assessed using gel fraction content determination, FTIR, ^1^H NMR, and energy-dispersive X-ray spectroscopy (EDX). SEM and DSC-based thermoporometry analyses demonstrated the formation of nanoporous structures after IL removal from CER networks, thus showing the effective role of IL as a porogen. Pore sizes varied from ~20 to ~180 nm with an average pore diameter of around 45–60 nm depending on the initial IL content. The thermal stability of nanoporous CER-based films was investigated by thermogravimetric analysis.

## Background

High crosslink density cyanate ester resins (CERs)—also known as polycyanurates (PCNs)—are commonly used in aerospace applications and electronic devices as high temperature polymer matrices [1–3]. The specific interest in these high performance polymers arises from their unique combination of intrinsic properties, including thermal, fire, radiation and chemical resistance, high tensile moduli (3.1–3.4 GPa) and glass transition temperatures (*T*_g_ > 220 °C), low dielectric constants (*ε* ~ 2.6–3.2), high adhesion to conductor metals and composites, and low water/moisture uptake [1, 2].

Ionic liquids (ILs) are organic salts that typically consist of bulky, asymmetric organic cations, and inorganic symmetric anions. Room-temperature ILs are defined as salts with melting points below or equal to room temperature [4, 5]. ILs have attracted widespread interest in polymer science due to their versatile properties, such as negligible saturated vapor pressure, wide liquid-state temperature range, nonflammability, incombustibility, high electrical conductivity, and good stability to oxidation [6–10]. They have progressively been used as solvents and catalysts for polymerization reactions [10] as well as additives in the design of polymer materials [11]. Their peculiar structure enables easy separation, recovery, and recycling of the catalyst from the reaction mixtures. In the case of membrane processes, ILs are being used in the design and modification of advanced materials that enable performance levels not typical of conventional materials [12]. Another application of ILs consists of their use as effective and reusable porogens in vinylic networks [13]. When ILs are used as porogenic solvents, during the in situ formation of polymer networks, chemically induced phase separation occurs. To act as efficient porogens, ILs have to possess: (i) high boiling temperature to avoid any premature evaporation, (ii) high thermal stability to remain unchanged up to the complete curing of the polymer networks, and (iii) easy extractability to be readily removed from the cured networks, thus affording porous thermosetting materials.

Porous polymeric materials have a large variety of applications in many areas as highly selective membranes, selective adsorbents and filters, porous electrodes for fuel cells, sensors or insulators, etc. [14]. Pioneering reports on the design of porous CERs were published by Hedrick and co-workers in the late 1990s [15–17]. Since 2008, two of our research groups have jointly developed various original approaches to nanoporous CER-based thermosetting films [18–23]. Two strategies relied on the use of oligo(ε-caprolactone) chains as porogens, which were removed from the synthesized CER networks by either extraction [18] or selective hydrolysis [19]. Alternatively, other pore generation methods involved: (i) the use of high-boiling temperature liquids, i.e., phthalates, as porogens [20, 21], (ii) the synthesis of CER networks with different degrees of cyanate group conversion, followed by the extraction of unreacted dicyanate monomer [22], and (iii) the irradiation of CER films by *α*-particles, followed by an alkaline etching to reveal the tracks created after bombing [23].

Recently, we have investigated the catalytic effect of ILs on the curing process of CERs and an acceleration effect has clearly been highlighted in the polycylotrimerization of dicyanate ester of bisphenol E in the presence of a specific ionic liquid [24]. To the best of our knowledge, ILs have not been used as porogens to generate porous CER thermosets so far. In the present work, novel nanoporous CER-based thermosetting films are engineered by using a room-temperature ionic liquid, namely, 1-heptylpyridinium tetrafluoroborate ([HPyr][BF_4_]), as a porogen and the effect of porogen content on the structure and properties of resulting porous CERs is examined.

## Methods

### Materials

1,1′-Bis(4-cyanatophenyl)ethane (dicyanate ester of bisphenol E (DCBE)), under the trade name Primaset™ LECy, was kindly supplied by Lonza (Basel, Switzerland) and was used as received. The following chemicals were used for the synthesis of the 1-heptylpyridinium tetrafluoroborate, [HPyr][BF_4_]: pyridine, 1-chloroheptane, ethyl acetate, hexane, tetrafluoroboric acid (48 wt% in H_2_O), methylene chloride, and sodium sulfate. The chemicals were provided by Fluka and were used as received.

### Ionic Liquid Synthesis

**Fig. 1:**
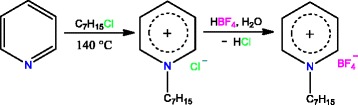
Synthetic route to ionic liquid [HPyr][BF_4_]

1-Heptylpyridinium tetrafluoroborate [HPyr][BF_4_] was synthesized using the following method. A mixture of dry pyridine:1-chloroheptane with a molar ratio 1.0:1.1 was heated at 140 °C for 20 h under stirring. A white solid product, i.e., 1-heptylpyridinium chloride, was obtained after cooling the reaction mixture to room temperature. It was purified by recrystallization from ethyl acetate/hexane mixture (1:1 vol/vol). 1-Heptylpyridinium chloride (50 g, 0.23 mol) was dissolved in 300 mL of water and 30 mL of tetrafluoroboric acid was added to the solution. The water immiscible layer of the ionic liquid [HPyr][BF_4_] formed was extracted with methylene chloride (3 × 150 mL), washed with water, and dried over sodium sulfate. The solvent was distilled off, and the resulting ionic liquid was dried under a reduced pressure of 1 mbar at 80 °C for 12 h. The synthetic route to the ionic liquid [HPyr][BF_4_] is depicted in Fig. 1.

### Preparation of CER-Based Films

**Fig. 2:**
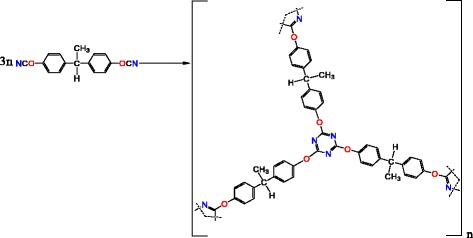
Scheme of CER network formation

DCBE was mixed with [HPyr][BF_4_] in a given ratio (the content of [HPyr][BF_4_] was equal to 20, 30, and 40 wt%), and the homogeneous mixtures were subjected to an ultrasonic bath at 60 °C for 30 min. These solutions were then poured into a PTFE-coated mold and cured over the temperature range from 25 to 250 °C with a heating rate of 0.5 °C min^−1^. The polycyclotrimerization of DCBE resulted in the formation of a CER network (see Fig. 2).

For generation of porous structure, the films obtained with a thickness around 100 μm were subjected to extraction with ethanol in a Soxhlet apparatus for 16 h. After extraction, the samples were dried up to a constant weight at 25 °C. The following codes were applied to the samples under investigation: CER_ext_, CER_20ext_, CER_30ext_, CER_40ext_, respectively, for the extracted CER sample synthesized without IL and for extracted CER samples synthesized in the presence of IL, where the subscripts indicate the initial content of [HPyr][BF_4_]. The code CER_40_ was applied to the nonextracted sample with a [HPyr][BF_4_] content of 40 wt%: it was used as a reference sample for the sake of comparison.

### Physico-chemical Techniques

Gel fraction contents of the CER-based networks were determined after Soxhlet extraction and drying up to constant weight. The experimental values of gel fraction contents, *w*_*g*(exp)_, were determined as the contents of insoluble part of the samples using Eq. 1:

1 $$ {w}_{g\left( \exp \right)}=\frac{m_2}{m_1}\cdot 100\% $$

where *m*_1_ and *m*_2_ stand for the mass of a dried sample before and after extraction, respectively.

The theoretical values of gel fraction contents were calculated with the conjecture that nonreactive [HPyr][BF_4_] was completely extracted from CER films using Eq. 2:

2 $$ {w}_{\mathrm{g}\left(\mathrm{theor}\right)\mathrm{CER}}={w}_{\mathrm{g}\left( \exp \right)\mathrm{CER}}-{w}_{\mathrm{g}\left[\mathrm{HPyr}\right]\left[{\mathrm{BF}}_4\right]} $$

where *w*_g(exp)CER_ and *w*_g[HPyr][BF4]_ stand for the experimental value of gel fraction content for pure CER (~100 wt%) and the initial [HPyr][BF_4_] content in the systems, respectively.

FTIR spectra were recorded on a Bruker Tensor 37 spectrometer between 4000 and 450 cm^−1^ using the attenuated total reflection (ATR) mode. For each spectrum, 32 consecutive scans with a resolution of 0.6 cm^−1^ were averaged.

^1^H NMR spectroscopy was conducted with a Bruker AV II spectrometer operating at a resonance frequency of 400 MHz. The spectra were recorded at room temperature using DMSO-*d*_6_ as an internal standard (*δ* = 2.5 ppm).

Scanning electron microscopy (SEM) analyses of the samples were performed on a MERLIN microscope from Zeiss equipped with Inlens and SE2 detectors using an accelerating voltage of 4 kV. Prior to analyses, the films were cryofractured and coated with a Pd/Au alloy (4 nm thickness) in a Cressington 208 HR sputter-coater. Energy-dispersive X-ray spectroscopy (EDX) was performed using a SSD X-Max detector of 50 mm^2^ from Oxford Instruments (127 eV for the *Kα* of Mn) coupled to the SEM equipment. To determine the main porosity characteristics derived from SEM data (i.e., pore sizes and pore size distributions), 1000 pores for each sample were at least evaluated using the ImageJ 1.48v software. Pores with area inferior to 20 nm^2^ and superior to 1.25 × 10^5^ nm^2^ were ignored to avoid counting of improbable values. Since pore circularity values revealed from ImageJ analysis varied from 0.80 to 0.90 (assuming that «0» corresponded to an infinitely elongated polygon and «1» was related to a perfect circle), pore diameters were calculated assuming circular pore shapes.

DSC-based thermoporometry was used as an independent quantitative technique for determining pore sizes and pore size distributions. The basic principles of this technique are well-known [25, 26]. In this study, thermoporometry was performed using water as a penetrating liquid. From the melting thermograms of water contained in the porous films, Eqs. 3–5 were applied to determine pore diameters (*D*_p_), pore size distributions (*dV*/*dR*_p_), and heat flow values Δ*H*(*T*), respectively:

3 $$ {D}_p(nm)=2\cdot \left(0.68-\frac{32.33}{T_m-{T}_{m0}}\right) $$

4 $$ d V/ d R\left( c{m}^3\cdot n{m}^{-1}\cdot {g}^{-1}\right)=\frac{dq/ d t\cdot {\left({T}_m-{T}_{m0}\right)}^2}{32.33\cdot \rho \cdot v\cdot m\cdot \varDelta H(T)} $$

5 $$ \varDelta H(T)\left( J\cdot {g}^{-1}\right)=332+11.39\cdot \left({T}_m-{T}_{m o}\right)+0.155\cdot {\left({T}_m-{T}_{m0}\right)}^2 $$

where *T*_m_ and *T*_m0_ are the melting temperatures of confined and bulk water, respectively; *dq*/*dt*, *ρ*, *ν*, *m*, and Δ*H*(*T*) are the heat flow recovered by DSC, the water density, the heating rate, the sample mass, and the melting enthalpy of water, correspondingly.

Due to the hydrophobicity of CER films, we resorted to an ethanol pretreatment in order to improve their hydrophilic character and favor the water penetration into the pores. Such a pretreatment using an organic solvent miscible with water, followed by its subsequent replacement by water, ensured pore accessibility to water. In addition, it was assumed that pore filling was predominant over the bulk polymer swelling in either ethanol or water, due to the high cross-link density of the CER network. The samples were first immersed to ethanol for 2 h, and then distilled water was gradually added to remove the ethanol. Afterwards, the samples were kept in pure distilled water for 2 weeks. After surface wiping, the melting thermograms were recorded using TA Instruments 2010 calorimeter under nitrogen atmosphere in temperature range from −50 to 5 °C at a heating rate of 1 °C min^−1^. The typical sample mass was about 10–15 mg.

Thermogravimetric analysis (TGA) measurements were performed using a Setaram SETSYS evolution 1750 thermobalance. Samples were heated in a platinum crucible from 20 to 700 °C at a heating rate of 10 °C min^−1^ under argon atmosphere.

## Results and Discussion

**Fig. 3:**
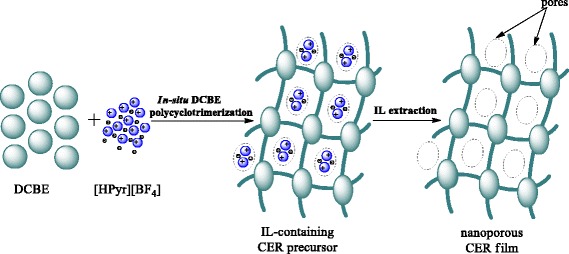
Representative scheme of CER formation in the presence of [HPyr][BF_4_] and subsequent pore formation

**Fig. 4:**
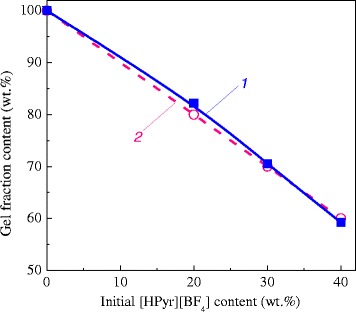
Experimental (*1*) and theoretical (*2*) values of gel fraction contents after extraction as a function of [HPyr][BF_4_] content

The generation of nanoporous thermosetting films was accomplished through the formation of CER-based thin films derived from the in situ polycyclotrimerization of DCBE in the presence of [HPyr][BF_4_] with further removal of the latter (Fig. 3). The gel fraction contents associated with CER samples were determined after ethanol extraction. Figure 4 clearly shows that the experimental and theoretical values of the gel fraction content nearly matched, thus strongly suggesting the completion of CER formation and [HPyr][BF_4_] extraction from CER-based networks, while confirming the chemical inertness of the ionic liquid towards CER. It should be noted that even a [HPyr][BF_4_] content as high as 40 wt% in the initial system did not hinder the formation of a highly cross-linked CER structure.

### Spectroscopic Analyses of Network Structure

**Fig. 5:**
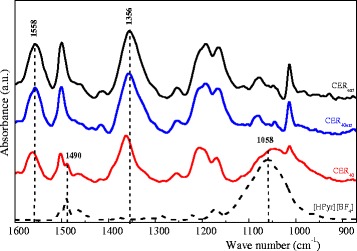
FTIR spectra of ionic liquid [HPyr][BF_4_] and typical CER networks before and after extraction

In order to evaluate the effect of [HPyr][BF_4_] on network structure and further confirm its chemical inertness to DCBE, FTIR analysis was performed. Figure 5 displays FTIR spectra of CER_ext_, CER_40ext_, CER_40_, and pure [HPyr][BF_4_]. The FTIR analysis of the CER_40ext_ sample (and of the other extracted CER samples, not shown here) demonstrated the presence of C = N–C and N–C–O stretching absorption bands of cyanurate repeating units at 1558 and 1356 cm^−1^, respectively, and did not indicate any stretching absorption bands of unreacted cyanate groups at 2272–2236 cm^−1^, thus corroborating the formation of CER network.

It should be pointed out that, for both the CER_40_ and the pure [HPyr][BF_4_], the band at 1490 cm^−1^ and the broad band with maximum at 1058 cm^−1^, corresponding to the stretching mode of pyridinium cation [27] and the asymmetric stretching of BF_4_^−^ anion of the ionic liquid, respectively, were observed. Logically, after [HPyr][BF_4_] extraction, such absorption bands disappeared from FTIR spectrum of the CER_40ext_ sample, while the intensities of the bands at 1558 and 1356 cm^−1^ did not change significantly. It is noteworthy that the well-defined bands in the region of 1100–1000 cm^−1^ in the spectra of CER_ext_ and CER_40ext_ corresponded to C–O–C bonds in the CER network structure. Consequently, FTIR analysis confirmed the chemical inertness of IL to the CER network and the efficient removal of [HPyr][BF_4_] from CER matrix.

**Fig. 6:**
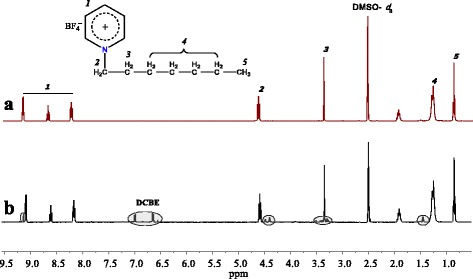
^1^H NMR spectra of [HPyr][BF_4_] (**a**) and sol fraction after CER_40_ extraction (**b**)

^1^H NMR spectra of [HPyr][BF_4_] and a typical sol fraction obtained after CER extraction are shown in Fig. 6. The resonance signals at 0.84, 1.28, 1.91, 3.34, and 4.60 ppm could be assigned to the protons from −C_7_H_15_, and the presence of protons from pyridinium ring could be observed with chemical shifts equal to 8.16, 8.60, and 9.08 ppm. Obviously enough, the ^1^H NMR spectrum of the sol fraction closely matched that of [HPyr][BF_4_], only traces of unreacted DCBE and/or soluble low molar mass cyanurate fragments nonincorporated into CER network being observed in the interval of 6.62–7.08 ppm. Once again, this spectroscopic analysis confirmed that [HPyr][BF_4_] was successfully removed from CER networks.

### SEM and EDX Analyses of CER-Based Films

**Fig. 7:**
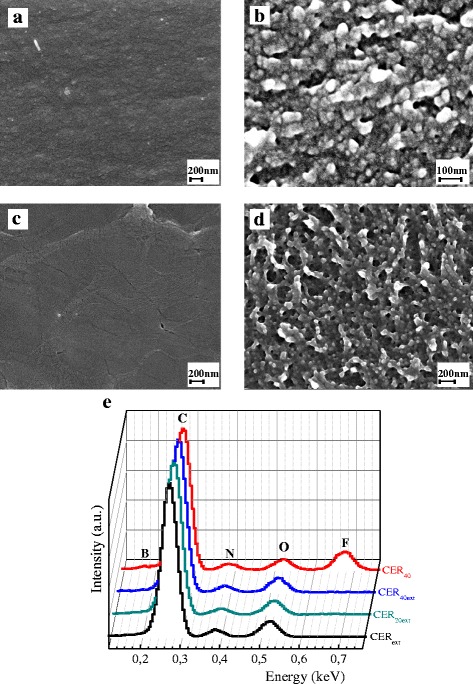
Typical SEM micrographs of CER-based samples: CER_ext_ (**a**), CER_20ext_ (**b**), CER_40_ (**c**), CER_40ext_ (**d**), and corresponding EDX spectra (**e**)

Typical SEM images of CER films before extraction of [HPyr][BF_4_] and after extraction of the latter are presented in Fig. 7. As it was expected, both CER_ext_ and CER_40_ samples (Fig. 7a, c, respectively) exhibited compact and nonporous structures, whereas CER_20ext_ and CER_40ext_ samples (Fig. 7b, d, respectively) displayed a nanoporous structure with pore diameters ranging from 25 to 170 nm, depending on their CER/IL composition. Pore sizes generally increased and pore size distributions widened when increasing the porogenic solvent (i.e., [HPyr][BF_4_]) content (Table 1) in the IL-filled CER precursors.

**Table 1:** Main porosity characteristics for nanoporous CER-based films

Porous films	SEM			DSC-based thermoporometry		
	Average pore diameter (nm)	Pore size distribution (nm)	Porosity ration	Average pore diameter (nm)	Pore size distribution (nm)	Total pore volume (cm^3^ g^−1^)
CER_20ext_	40	~25–100	0.18	45	~20–105	0.037
CER_30ext_	60	~25–165	0.30	60	~20–175	0.120
CER_40ext_	65	~25–170	0.39	60	~20–180	0.124

**Fig. 8:**
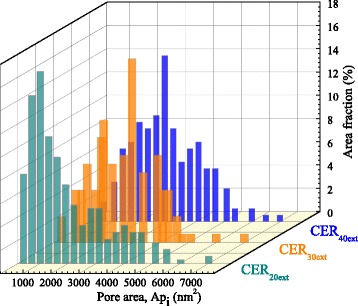
Pore area distributions derived from SEM data for the nanoporous CER-based samples

The micrographs obtained were carefully analyzed using the ImageJ software. Most of pore area fractions ranged from 500 to 5000 nm^2^ (Fig. 8) that corresponded to pore diameters (*D*_p_) from ~25 to 80 nm. The quantity of larger pores (with pore area higher than 5000 nm^2^, i.e., *D*_p_ > 80 nm) turned out to be negligible. The values of average pore diameters were found to be around 40, 60, and 65 nm for CER_20ext_, CER_30ext_, and CER_40ext_, respectively (Table 1). It is noteworthy that the porosity ratio values as determined from SEM data were in excellent agreement with expected values, considering the complete removal of IL initial content.

Besides SEM micrographs, Fig. 7 also shows corresponding EDX spectra. As expected, the absence of B and F elements of [HPyr][BF_4_] was observed in the porous samples studied, which confirmed the complete extraction of IL from CER/IL precursor samples. Table 2 summarizes the experimental and theoretical values of element contents in the CER-based samples under study. Interestingly, both sets of values were in good agreement.

**Table 2:** Experimental and theoretical values of element contents in typical CER-based samples

Samples	Element contents (wt%)									
	Experimental (EDX)					Theoretical (calculated)				
	C	N	O	F	B	C	N	O	F	B
CER_ext_	76.0	12.0	12.0	0	0	76.2	11.1	12.7	0	0
CER_20ext_	75.8	12.1	12.1	0	0	76.2	11.1	12.7	0	0
CER_30ext_	76.8	11.5	11.7	0	0	76.2	11.1	12.7	0	0
CER_40ext_	77.3	11.7	11.0	0	0	76.2	11.1	12.7	0	0
CER_40_	67.1	9.0	7.1	14.4	2.4	69.3	9.0	7.7	12.1	1.8

### Investigation of Nanoporous CER-Based Films by DSC-Based Thermoporometry

**Fig. 9:**
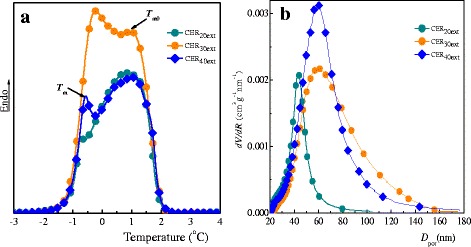
DSC melting thermograms of water confined within the pores of nanoporous CERs (**a**) and corresponding pore size distribution profiles (**b**)

The melting thermograms of water in nanoporous CER samples in the temperature region between −3 and 4 °C as well as the corresponding profiles of pore size distributions are given in Fig. 9a, b, respectively. In the thermograms of CER samples, two endothermic peaks were detected: one with a maximum, *T*_m_, between −2 and 0 °C corresponding to the melting of water constrained within the pores of the films, and a second one with a maximum, *T*_m0_, between 0 and 2 °C related to the melting of bulk water (Fig. 9a). It was found that pore size distributions for the porous CER-based films under investigation were in the range of ~20–180 nm (Fig. 9b) and their average pore diameters were around 45–60 nm, depending on the initial IL content in the CER precursors (Table 1). It is noteworthy that an increase in the [HPyr][BF_4_] content resulted in an increase in pore diameters and a broadening of pore size distributions, along with increasing pore volumes. These results were in close agreement with those obtained from SEM analysis. Minor discrepancies between the pore characteristics determined by both techniques could be explained by a difference between real pore shapes of CER structures and circular ones used for the mathematical data processing in SEM analysis.

### Thermal Stability of CER-Based Films by TGA

**Fig. 10:**
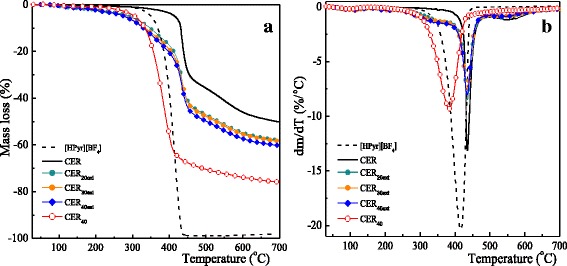
Mass loss (**a**) and corresponding derivative (**b**) curves as determined by TGA for [HPyr][BF_4_] and typical CER-based films

The influence of [HPyr][BF_4_] on the thermal stability of nanoporous CER networks was investigated by TGA. Mass loss and corresponding derivative curves are presented in Fig. 10, and the main corresponding data are summarized in Table 3. For the neat CER sample, the first step of the intensive mass loss was observed in the temperature range of ~390–490 °C, which was associated with the degradation of the skeleton of cross-linked CER network, and the second step was observed at higher temperatures with a small mass loss. For pure [HPyr][BF_4_], we could observe a single degradation stage in the temperature interval ranging from ~350 to 438 °C with an intense mass loss value of about 94 wt%.

**Table 3:** TGA data obtained for CER-based networks and pure [HPyr][BF_4_]

Samples	*T*_d1_^a^ (°C)	*T*_dmax_^b^ (°C)	*T*_d50%_^c^ (°C)	Mass loss at *T*_dmax_ (%)	Char residue (wt%)
CER	425	435	694	16	50
CER_20ext_	399	435	538	32	42
CER_30ext_	396	434	528	33	42
CER_40ext_	395	435	503	35	40
CER_40_	340	385	393	42	25
[HPyr][BF_4_]	376	416	407	67	2

^a^Onset temperature of intensive degradation as determined by value for intersection of tangents to curve at the first inflection point

^b^Temperature value of maximal degradation rate

^c^Temperature values for a 50% mass loss

In contrast to pure CER, the thermal decomposition of nanoporous CER films was more complex and involved more stages, especially in the lower temperature range. A slight initial mass loss of about 2–5 wt% below 280 °C might arise from the removal of entrapped moisture within the CER network. The first decomposition step for CER_20ext_, CER_30ext_, and CER_40ext_ really occurred in the temperature range of 285–395 °C with a mass loss of ~14–17 wt% corresponding to the degradation of porous and defective network regions of CER structures. Near 400 °C, the onset temperature of intensive degradation with higher mass loss (~29–30 wt%) then occurred, which could be attributed to the destruction of triazine cycles of CER skeleton. The overall decomposition approximately led to 40–42 wt% char residues. Surprisingly, the thermal stability of the nanoporous CER-based networks decreased compared to that of pure CER, although they had the same chemical structure: the temperature values for onset of intensive degradation (*T*_d1_) and 50% mass loss (*T*_d50%_) decreased, respectively, from 425 and 694 °C for the CER sample to 395 and 503 °C for the CER_40ext_ sample (Table 3). The higher the initial [HPyr][BF_4_] content in the CER precursors, the lower the thermal stability of the nanoporous films obtained. Nevertheless, the temperatures of maximum mass loss (*T*_dmax_) are nearly identical for the CER, CER_20ext_, CER_30ext_, and CER_40ext_ films. Interestingly, the presence of IL in the CER_40_ sample led to a significant reduction of *T*_dmax_ by 50 °C and one such film degraded in a single step. One could suppose that one such strong dilution (40 wt% of IL) hindered the DCBE polycyclotrimerization, as the probability of the elementary reaction step might decrease, i.e., the reaction of three cyanate groups together to afford the formation of cyanurate rings. The existence of molecules of DCBE monomer or other intermediate oligomeric molecules, which were not incorporated into the CER network (as confirmed by ^1^H NMR spectrum of the sol fraction, see Fig. 6), along with IL, might drastically decrease the *T*_dmax_ value of CER_40_ sample. After extraction of all the soluble fragments, the final nanoporous CER_40ext_ sample was characterized by a high *T*_dmax_ value comparable to that of the other nanoporous materials obtained (*T*_dmax_ = 435 °C).

Removing IL from CER precursory networks afforded nanoporous films, which degraded in two steps corresponding to the destruction of the defective CER network at lower temperatures and the degradation of the regularly cross-linked network regions with *T*_dmax_ equal to that of nonporous CER analog. Consequently, applying [HPyr][BF_4_] as a porogen reduced the thermal stability of resulting nanoporous films compared to that of neat CER to some extent, probably due to the formation of less regular CER structures.

## Conclusions

Novel nanoporous film materials based on thermostable polycyanurates generated in situ by polycyclotrimerization of DCBE in the presence of ionic liquid [HPyr][BF_4_] have been developed. Nanoporous CER-based films were obtained by extraction of the ionic liquid from CER networks. Complete IL removal was confirmed by determination of gel fraction contents, FTIR, ^1^H NMR, and EDX spectroscopic analyses. SEM and DSC-based thermoporometry were used as complementary techniques for nanopore characterization. Depending on the IL porogen content, the average pore diameter values were found in the range of 45–60 nm with pore size distributions of ~20–180 nm. It is also noteworthy that an increase in the [HPyr][BF_4_] content resulted in increasing pore diameters and broader pore size distributions. The TGA curves showed high thermal stability of the nanoporous films obtained with an onset decomposition temperature near 300 °C. It should be stressed that the synthesis of CERs in the presence of IL was carried out without using any additional solvent or specific catalyst, the ionic liquid being highly thermostable and potentially being utilized repeatedly.

## Abbreviations

[HPyr][BF_4_]: 1-Heptylpyridinium tetrafluoroborate
CER: Cyanate ester resin
DCBE: Dicyanate ester of bisphenol E
IL: Ionic liquid
